# Renal angiomyolipoma: a radiological classification and update on recent developments in diagnosis and management

**DOI:** 10.1007/s00261-014-0083-3

**Published:** 2014-02-07

**Authors:** Masahiro Jinzaki, Stuart G. Silverman, Hirotaka Akita, Yoji Nagashima, Shuji Mikami, Mototsugu Oya

**Affiliations:** 1 Department of Diagnostic Radiology, Keio University School of Medicine, 35 Shinanomachi, Shinjuku-ku, Tokyo 160-8582 Japan; 2 Department of Radiology, Brigham and Women’s Hospital, 75 Francis St., Boston, MA 02115 USA; 3 Department of Molecular Pathology, Yokohama City University Graduate School of Medicine, 3-9, Fukuura, Kanazawa-ku, Yokohama, Japan; 4 Division of Diagnostic Pathology, Keio University Hosoital, 35 Shinanomachi, Shinjuku-ku, Tokyo Japan; 5 Department of Urology, Keio University School of Medicine, 35 Shinanomachi, Shinjuku-ku, Tokyo, Japan

**Keywords:** Angiomyolipoma, Perivascular epithelioid cell tumors, PEComa, Renal cell carcinoma, Fat poor AML, AML with minimal fat

## Abstract

Angiomyolipoma is the most common benign solid renal neoplasm observed in clinical practice. Once thought to be a hamartoma and almost always diagnosed by the imaged-based detection of fat, angiomyolipomas are now known to consist of a heterogeneous group of neoplasms. Although all are considered perivascular epithelioid cell tumors, many display different pathology, imaging features, and clinical behavior. The importance of understanding this group of neoplasms is emphasized by the fact that many types of angiomyolipoma contain little to no fat, and despite being benign, sometimes escape a pre-operative diagnosis. These types of angiomyolipomas can all be considered when encountering a renal mass that is both hyperattenuating relative to renal parenchyma on unenhanced CT and T2-hypointense, features that reflect their predominant smooth muscle component. We review recent developments and provide a radiological classification of angiomyolipomas that helps physicians understand the various types and learn how to both diagnose and manage them.

Angiomyolipoma is a solid tumor that is encountered commonly in the kidney in clinical practice [1, 2]. Angiomyolipoma is typically a solid “triphasic” tumor composed of varying amounts of three elements: dysmorphic blood vessels, smooth muscle components, and mature adipose tissue [1]. Because most angiomyolipomas contain substantial amounts of adipose tissue, it is usually diagnosed using CT or MRI by identifying imaging features of fat cells in the mass [2]. Those that are able to be diagnosed using imaging have been called “classic” angiomyolipomas [2]. While 80% of angiomyolipomas are sporadic and most of them inconsequential, approximately 20% are associated with tuberous sclerosis complex (TSC) [10]. Angiomyolipomas may be found also in patients with lymphangioleiomyomatosis (LAM) [11, 12].

Recent developments have added to our understanding of renal angiomyolipoma. First, although once considered a hamartoma, and more recently a choristoma [2, 3], angiomyolipoma is now considered among the family of perivascular epithelioid cell tumors (PEComa) [13]. In addition, recent developments have revealed that there are different types of renal angiomyolipoma; collectively, they compose a heterogeneous group of neoplasms with variable pathology, radiology, and clinical behavior. For example, in addition to the classic angiomyolipoma, it is now recognized that some triphasic angiomyolipomas, so-called fat poor angiomyolipoma, contain only small amounts of fat cells [4, 5], too few to be detected with imaging, and are sometimes mistaken for renal cancers [6]. A recently described pathological entity called angiomyolipoma with epithelial cysts also contains few or no fat cells, and is among the group of fat poor angiomyolipomas [7, 8]. Although angiomyolipoma is usually benign, a rare, potentially malignant epithelioid angiomyolipoma is listed separately in the most recent World Health Organization (WHO) classification of renal tumors [1, 9].

Understanding recent developments and both the radiological and pathological differences between the types of angiomyolipoma is important both in clinical practice and for future research. In this review, we present a radiological classification of angiomyolipoma based on specific imaging features, and incorporating recent developments (Table 1). Our classification is consistent with the WHO classification of angiomyolipomas [1]. However, the current WHO classification is based on findings at pathology alone, and was derived not with the intent of describing the varied radiological appearances of angiomyolipomas. Our radiologic classification provides what is currently known about both the pathology and the radiology of renal angiomyolipomas. Knowledge of the imaging appearance of the various types, particularly as it relates to both their pathology and clinical behavior, can assist in the diagnosis and management of these neoplasms.

**Table 1:** Radiological classification of renal angiomyolipoma

	Frequency	CT	MRI	US	Amount of fat cells	Clinical behavior	Management
Sporadic	80%						
Triphasic AML							
Classic AML	Common	Fat attenuation and CS MRI^a^	Signal loss on FS MRI	Markedly hyperechoic	Abundant	Benign	Observation
Fat poor AML	Uncommon	No evidence of fat at unenhanced CT^b^					
Hyperattenuating AML	Approximately 4.5% of all AMLs	Hyperattenuating (>45 HU), homogenously enhancing	T2-hypointense	Isoechoic	Few or none	Benign	Biopsy followed by observation
		Variable enhancement pattern	No signal loss on CS MRI				
			No signal loss on FS MRI				
Isoattenuating AML	Rare	Isoattenuating (−10 to −45 HU)	T2-hyopointense	Slightly hyperechoic	Scattered	Benign	Biopsy followed by observation
		Variable enhancement pattern	Signal loss on CS MRI				
AML with epithelial cysts	Rare	Hyperattenuating with cysts or multilocular cystic	+/−Signal loss on FS MRI^c^	Unknown	Few or none	Benign	Biopsy followed by observation
			T2-hypointense in solid component				
Epithelioid AML	Rare	Hyperattenuating, (>45 HU) heterogeneously enhancing or multicystic	T2-hypointense in solid component	Unknown	Few or none	Potentially malignant	Resection or mTOR inhibitor
		Hemorrhage may be massive					
		Variable enhancement pattern					
Syndromic	20%						
AML in tuberous sclerosis complex		Variable	Variable	Variable	Any amount	Both benign and potentially malignant^d^	Relative to sporadic types, AML in TSC are, more likely to need treatment including resection, embolization, ablation or mTOR inhibitor
AML in lymphangioleiomyomatosis		Variable	Variable	Variable	Any amount	Both benign and potentially malignant^d^	Similar to sporadic types, but mTOR inhibitor can be one choice

Attenuation values are approximations

AML, angiomyolipomas; FS, frequency-selective fat suppression; CS, chemical shift

^a^Some classic angiomyolipomas demonstrate no chemical shift suppression (image voxels contain virtually all fat)

^b^When thin (1.5–3 mm) sections are used

^c^Signal loss on FS MRI may or may not be present

^d^Epithelioid angiomyolipomas may occur in patients with tuberous sclerosis complex and lymphangioleiomyomatosis

## Angiomyolipoma: a member of the family of perivascular epithelioid cell tumors

The term perivascular epithelioid cell tumors (PEComa) was introduced by Zamboni et al. in 1996 [20]. In 2002 and 2003, two monographs published under the auspices of the WHO recognized a family of neoplasms with perivascular epithelioid cell (PEC) differentiation and coined the term, “PEComa” [14]. PEComas are mesenchymal neoplasms composed of nests and sheets of predominantly epithelioid and some spindle cells that are associated with blood vessel walls, and the perivascular epithelioid cell or “PEC” (a cell that has no known normal tissue counterpart). Nearly, all PEComas show immunoreactivity for both melanocytic [Human melanasome B (HMB)-45 and/or melan-A] and smooth muscle (smooth muscle actin (SMA) and/or desmin) markers [15]. This family of neoplasms is a group of morphologically and immunophenotypically similar lesions that can arise at a variety of visceral and soft tissue sites. A subset of the smooth muscle cells in angiomyolipomas are epithelioid in appearance and often arranged around blood vessels, a feature that is characteristic of a PEC [3, 15]. Thus, angiomyolipomas belong to the PEComa family that includes also pulmonary lymphangioleiomyomatosis and clear cell “sugar” tumor of the lung [1, 3, 13–16]. PEComas are related to the genetic alterations found in patients with TSC, an autosomal dominant genetic disease due to losses of TSC1 (9q34) or TSC2 (16p13.3) genes that may have a role in the regulation of the Rheb/mTOR/p70S6K pathway, which increase protein synthesis and consequently, cell growth [16].

## Triphasic angiomyolipoma

Triphasic angiomyolipoma is a benign mesenchmal tumor composed of varying amounts of dysmorphic blood vessels, smooth muscle components, and mature adipose tissue [1]. It occurs sporadically in less than 0.2% of people [2], typically during the 4th to 6th decade of life, and exhibits a female preponderance [3]. Triphasic angiomyolipoma can be divided radiologically into “classic” and “fat poor subtypes” [2].

## Classic angiomyolipoma

**Fig. 1:**
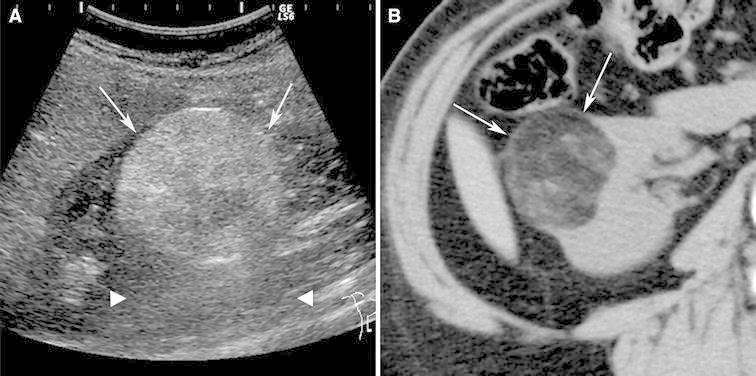
Angiomyolipoma, classic type, in a 61-year-old woman. Ultrasound (**A**) shows a 4.2-cm right renal mass (*arrows*) that is markedly hyperechoic relative to renal parenchyma and accompanied by acoustic shadowing (*arrow heads*). Transverse, unenhanced CT image (5-mm sections) shows a right renal mass (*arrows*) with fat attenuation (−60 HU).

The hallmark pathology feature of classic angiomyolipoma is abundant fat. The term “fat” in this context is used to refer to one or more fat cells. On ultrasound, classic angiomyolipoma is almost always markedly hyperechoic to renal parenchyma, often as hyperechoic as renal sinus fat [17, 18] (Fig. 1). Since renal cell carcinoma (RCC) also may be hyperechoic, ultrasound cannot be used alone to diagnose a classic angiomyolipoma. An anechoic rim and intratumoral cysts seen in 73% and 31%, respectively, in RCC smaller than 3 cm are suggestive of RCC because these findings are rarely seen in angiomyolipoma [18]. In addition, acoustic shadowing is seen in 21–33% of angiomyolipomas smaller than 3 cm [17, 18] (Fig. 1) and not typically seen in RCC. In spite of these characteristic findings, a confident diagnosis of a classic angiomyolipoma requires the identification of fat using CT or MRI.

**Fig. 2:**
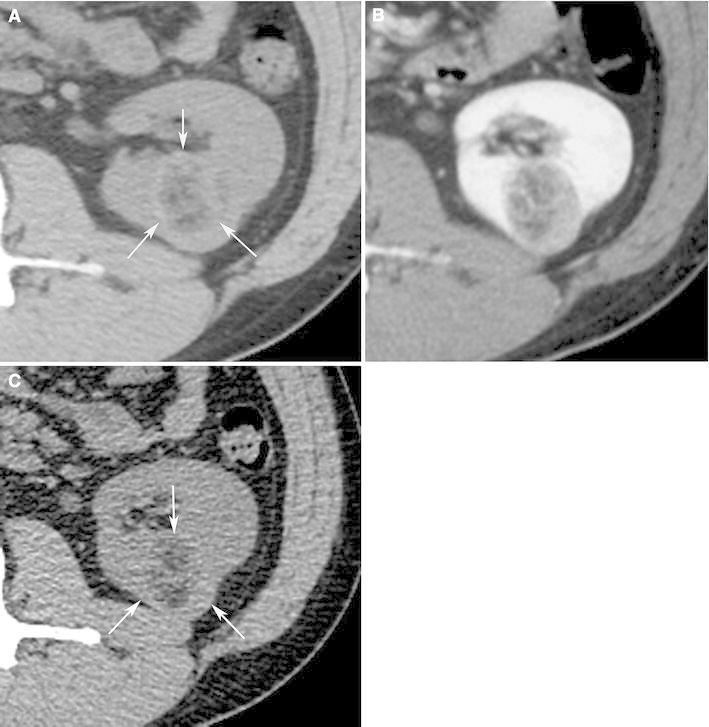
Angiomyolipoma, classic type, with small amount of fat in a 40-year-old man. Transverse, unenhanced CT (**A**) and enhanced (**B**) images (both using 5-mm sections) shows a 3.4-cm left renal mass (*arrows*) attenuation values that were measured on unenhanced images were all higher than −10 HU. When 1.5-mm sections were reconstructed (**C**), an attenuation of −25 HU was obtained and the diagnosis of angiomyolipoma was made. Fat attenuation was identified only on unenhanced CT.

The image-based detection of fat often begins with CT [19]. On unenhanced CT, the presence of regions of interest (ROI)-containing attenuations less than −10 HU allows the confident identification of fat (Fig. 1) [19–21]. The CT appearance of a classic angiomyolipoma varies due to variable amounts of fat, blood vessels, and smooth muscle components of the neoplasm. These neoplasms do not contain smooth muscle; they reveal smooth muscle-like cells (hence the term “components”) which typically stain positive for HMB-45 and smooth muscle markers. When evaluating angiomyolipomas with a small amount of fat with CT, the acquisition of thin (1.5–3 mm) sections and obtaining attenuation measurements using small ROI or even pixel values may be necessary to detect fat that otherwise would not be detected because of partial volume-averaging [20, 22, 23] (Fig. 2). In addition, intratumoral hemorrhage may occur, particularly in tumors larger than 4 cm; the high attenuation blood may mask the fat, particularly if there is a small amount, and lead to misdiagnosing a classic angiomyolipoma as a renal cancer [24].

Other entities may contain fat and thus regions of fat attenuation. Liposarcoma may contain fat but rarely originates in the kidney. The major imaging feature that helps distinguish angiomyolipoma from liposarcoma is the “beak sign” or a small divot at the interface of the mass with the kidney, often accompanied by a feeding vessel; both signs indicate a renal origin [25–27]. A fat-containing mass that originates in the kidney is likely an angiomyolipoma rather than liposarcoma. Identification of enlarged or bridging vessels, aneurysms, and perinephric hematomas are additional imaging features of angiomyolipoma that are rarely seen with liposarcoma [25, 27]. Although rare, Wilms’ tumors and RCC both may contain fat. Wilms’ tumors are uncommon in adults [28]. Most reported cases of fat-containing RCC also contain calcifications; the pathogenesis is thought to be due to osseous metaplasia leading to marrow fat formation [29, 30]. Since angiomyolipoma rarely contains calcifications, the presence of calcification and fat should raise the possibility of RCC. As a result, a confident diagnosis of an angiomyolipoma requires that a fat-containing mass not contain calcification. However, non-calcified, fat-containing RCC has been reported; fat attenuations in these cases are thought to be due to lipid-laden macrophages and cholesterol necrosis [31, 32]. Despite these rare exceptions, it is considered appropriate to diagnose an angiomyolipoma with confidence when a non-calcified, fat-containing renal mass is encountered in an adult [6]. Other lesions that may contain fat include teratomas, but these also usually contain calcifications. Finally, some non-fat containing lesions may appear to contain fat by engulfing sinus or perinephric fat [33]. RCC that is treated with ablation may appear to contain fat because the effects of the ablation often include a margin that contains peritumoral fat [34].

MRI can be used to detect fat cells and diagnose angiomyolipoma also. Current MR imaging methods cannot be used to differentiate fat (or lipid) in fat cells from fat in the cytoplasm of other types of cells. The diagnosis of the presence of fat is based on the amount of intra-voxel fat, not necessarily the cell type. For example, frequency selective (FS) fat suppression generally indicates the presence of fat cells (adipose tissue) [35]. However, when there are cells containing no fat (only water) in the same voxel as fat cells or other cells containing intracystoplasmic fat, the amount of fat is generally insufficient to be detected with the FS fat suppression technique [36, 37]. On the other hand, chemical shift suppression can be seen when there is a small amount of fat; this technique relates to the fact that signal suppression occurs when voxels contain both fat and water. However, chemical suppression MRI cannot be used to distinguish fat cells from other types of cells that contain intracystoplasmic fat. Therefore, chemical suppression may not be able to be used to distinguish small amounts of fat cells in an angiomyolipoma from cells containing intracytoplasmic fat in clear cell RCC [38]. The pattern of suppression on opposed phase images appears to be important. When the signal loss appears at the border of the mass and the renal parenchyma, it has been called an “india ink artifact” and is indicative of an angiomyolipoma [37]. However, renal masses that demonstrate chemical shift suppression throughout the mass (but do not suppress on FS fat suppression techniques) can be due to clear cell RCC or angiomyolipoma with small amounts of fat cells dispersed throughout the mass. In our experience, such masses are more often clear cell RCC, but this deserves further study.

The management of classic angiomyolipoma is conservative; most do not grow and remain asymptomatic. However, some grow slowly, typically at a rate of 5% per year or 0.19 cm per year [39, 40]. Some angiomyolipomas, particularly those larger than 4 cm, may bleed spontaneously [39, 40]. Thus, although all classic angiomyolipomas are benign, some form of radiological follow-up may be indicated, even when they are asymptomatic. Oesterling et al. [41] proposed an angiomyolipoma management algorithm based on tumor size and symptoms. For small (≤4 cm) asymptomatic tumors, observation with US every 12 months is suggested. If the tumor is small and the patient symptomatic, treatment with arterial embolization or partial nephrectomy can be considered but observation is often favored in clinical practice. For symptomatic patients with large tumors, particularly those whose tumors have bled, treatment is generally recommended. For large tumors in asymptomatic patients, observation with CT or US is recommended. However, despite these recommendations, there is no consensus as to which asymptomatic angiomyolipomas, if any, need imaging surveillance. To aid in which approach is best, and to better distinguish those patients at risk for hemorrhage, an aneurysm size of 5 mm or larger has been found to predict bleeding with a 100% sensitivity and 86% specificity, whereas a tumor size of 4 cm or larger resulted in sensitivity and specificity of 100 and 38%, respectively [42]. Others have classified angiomyolipomas larger than 4 cm into three groups based on their vascularity at angiography [43]. In this study, “minimal vascularity” was defined as having few, small, stretched pathological vessels, “moderate vascularity” as abundant, medium-size, tortuous vessels with or without small aneurysms (<5 mm), and “marked vascularity” as multiple, large tortuous vessels with/without large aneurysms (>5 mm) [43]. Angiomyolipomas larger than 4 cm with minimal vascularity were significantly less likely to require intervention due to bleeding (14.3%) than those with marked vascularity (50%) [43]. Renal arterial embolization and partial nephrectomy are typically are used to treat renal angiomyolipomas. However, renal arterial embolization carries a complication rate of 10% [44] with a 30% risk of post-embolization syndrome due to an inflammatory response to necrotic tissue [45], and partial nephrectomy carries a complication rate of 5–23% [46]. Recently, ablation, using transarterial ethanol, or percutaneous ablation (using cryoablation or radiofrequency) has been introduced as a third option, but more experience will be needed to establish its role [47–50].

## Fat poor angiomyolipoma

Some triphasic angiomyolipomas contain too little fat (i.e., too few fat cells) to be detected with unenhanced CT; some have been diagnosed pre-operatively as RCC and inadvertently removed at surgery [6]. These subtypes are now collectively referred to as “fat poor angiomyolipomas” [2]. By definition, these lesions do not reveal fat at unenhanced CT, even when thin (1.5–3 mm) sections are used. In 1997, the term “angiomyolipoma with minimal fat” was introduced to describe angiomyolipomas that were hyperattenuating relative to renal parenchyma on unenhanced CT, homogeneously enhancing, and at pathology were composed almost entirely of a smooth muscle component and little to no fat [5]. However, this term was used in subsequent studies to describe all angiomyolipomas in which fat could not be detected with unenhanced CT [51–53]. We now know that fat poor angiomyolipomas can be hyperattenuating or isoattenuating. Therefore, lesions that were described as angiomyolipomas with minimal fat represented only a subset of fat poor angiomyolipomas [6]. Furthermore, several additional studies have used other terms to describe fat poor angiomyolipomas, including “angiomyolipoma without visible fat on unenhanced CT”, “minimal fat angiomyolipoma” and “lipid-poor angiomyolipoma” [54–57]. For the most part, the lesions in these studies represented fat poor angiomyolipomas, but the investigators use of different terms may have suggested to some readers that the lesions were different. To reduce this confusion, and potential communication and documentation errors regarding angiomyolipomas that do not reveal evidence of fat cells on unenhanced CT, we believe the term “fat poor angiomyolipoma” is the most appropriate [2]. These lesions’ pathology explains the rationale for the use of this term and also explains why there are multiple subtypes of fat poor angiomyolipomas.

It has been difficult to define at pathology the amount of fat below which the mass would be considered a “fat poor angiomyolipoma”. All such masses have insufficient amounts of fat to be detected by imaging but the detection of fat depends both on the amount of fat and its distribution in the mass. An angiomyolipoma may be overall composed of a certain amount of fat, but the distribution of fat may be focal, scattered, or diffuse. One proposed definition of a fat poor angiomyolipoma is that the lesion contain no more than 25% fat cells per high powered field (hpf) [4]. However, with this definition, if all the fat cells were located in one region of the mass, the fat may be detectable with imaging even though it is “fat poor” according to this pathology definition. On the other hand, a pathology definition that includes masses with a larger percentage of fat cells may consist of masses that contain fat cells that are scattered and diffusely distributed and not detectable with imaging. Hence this hypothetical lesion would not be “fat poor” by pathology, yet the fat would not be detectable with imaging. Despite this limitation, it has been found that most fat poor angiomyolipomas contain less than 25% fat cells/hpf, and hence this definition reported in the pathology literature is one that makes the most sense to adopt in a radiological classification [4]. Dividing fat poor angiomyolipomas into three subtypes—hyperattenuating and isoattenuating angiomyolipomas, and angiomyolipoma with epithelial cysts—addresses the relationship of the amount of fat cells and their distribution in the mass [4].

### Hyperattenuating angiomyolipoma

Hyperattenuating angiomyolipoma is now the preferred term to describe a lesion that was originally described as an “angiomyolipoma with minimal fat”; these lesions represent approximately 4–5% of all angiomyolipomas [5]. They are typically small and average 3 cm in diameter [5, 58, 59]. At pathology, they generally contain only 4% (range, 3–10%) fat cells [58]. The abundant smooth muscle component generally stains positive for HMB-45 antigen and smooth muscle markers [5].

**Fig. 3:**
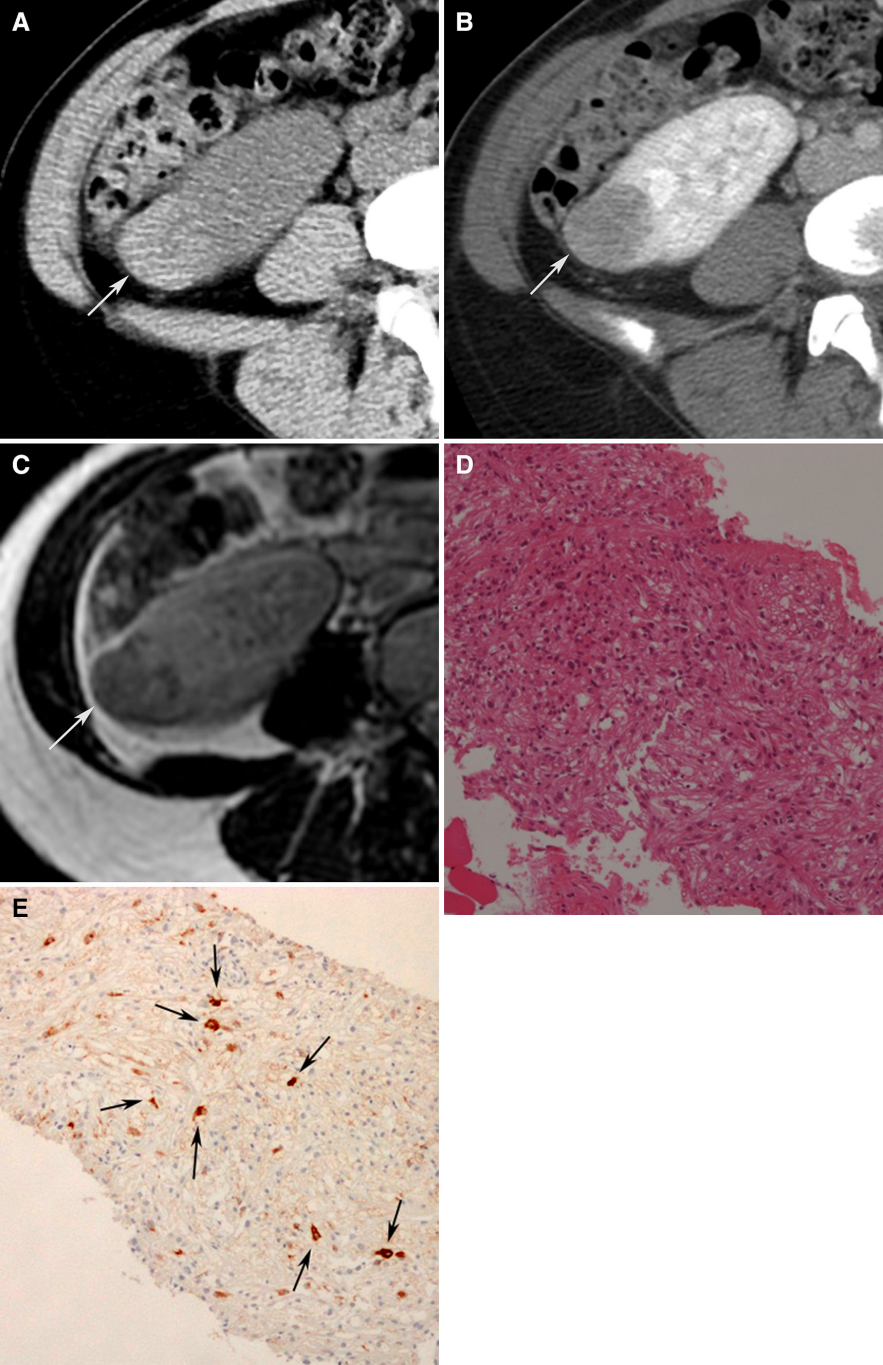
Angiomyolipoma, fat poor type, hyperattenuating subtype in a 39-year-old woman. Transverse, unenhanced CT (5-mm sections) demonstrates a 2.9-cm hyperattenuating (47 HU) right renal mass (*arrows*) with no regions that measured less than 30 HU (**A**). The mass-enhanced homogenously at CT (**B**) and was hypointense on transverse T2-weighted image (fast spin-echo, TR:2400, TE:94) (**C**). Biopsy specimen showed smooth muscle component (hematoxylin–eosin stain; original magnification, ×100.) (**D**), and was positive for HMB-45 (**E**: *arrows*).

Because of the abundant smooth muscle component, all hyperattenuating angiomyolipomas are hyperattenuating relative to renal parenchyma on unenhanced CT (usually more than 45 HU) similar to smooth muscle elsewhere, and typically homogeneously enhancing on CT [5, 58] (Fig. 3). On MRI, they behave similarly to smooth muscle and are T1-hypointense, T2-hypointense, and like CT, often enhance homogeneously [5, 58] (Fig. 3). Typically, there are no regions that show signal loss on fat-suppressed pulse sequences and no chemical shift suppression. On ultrasound, they are usually homogeneously isoechoic, similar to smooth muscle elsewhere [5, 59], although one study reported they may be hyperechoic [58]. In addition to angiomyolipoma, hyperattenuating, homogeneously enhancing renal masses can be the result of other entities including RCC (typically the papillary type), oncocytoma, lymphoma, metanephric adenoma, leiomyoma, and metastases [6, 60]. In the absence of a malignant tumor elsewhere, the likely causes of a hyperattenuating renal mass are RCC, hyperattenuating angiomyolipoma, and oncocytoma. In one study, only 2% of RCC were hyperattenuating and homogeneously enhancing, and none was isoechoic [5]. Therefore, when encountering a small (less than or equal to 3 cm) renal mass that is hyperattenuating on unenhanced CT and homogeneously enhancing, a hyperattenuating angiomyolipoma should be considered, particularly, if it is isoechoic on US or T2-hypointense on MRI [5, 6, 60, 61]. Percutaneous biopsy is recommended to provide a confident diagnosis of an angiomyolipoma [5, 6, 60, 61] (Fig. 3).

### Isoattenuating angiomyolipoma

**Fig. 4:**
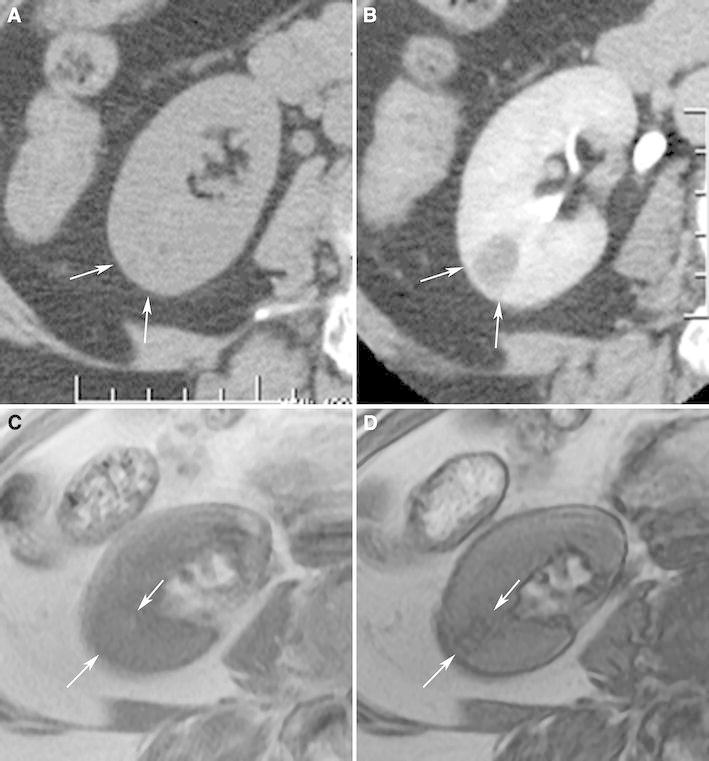

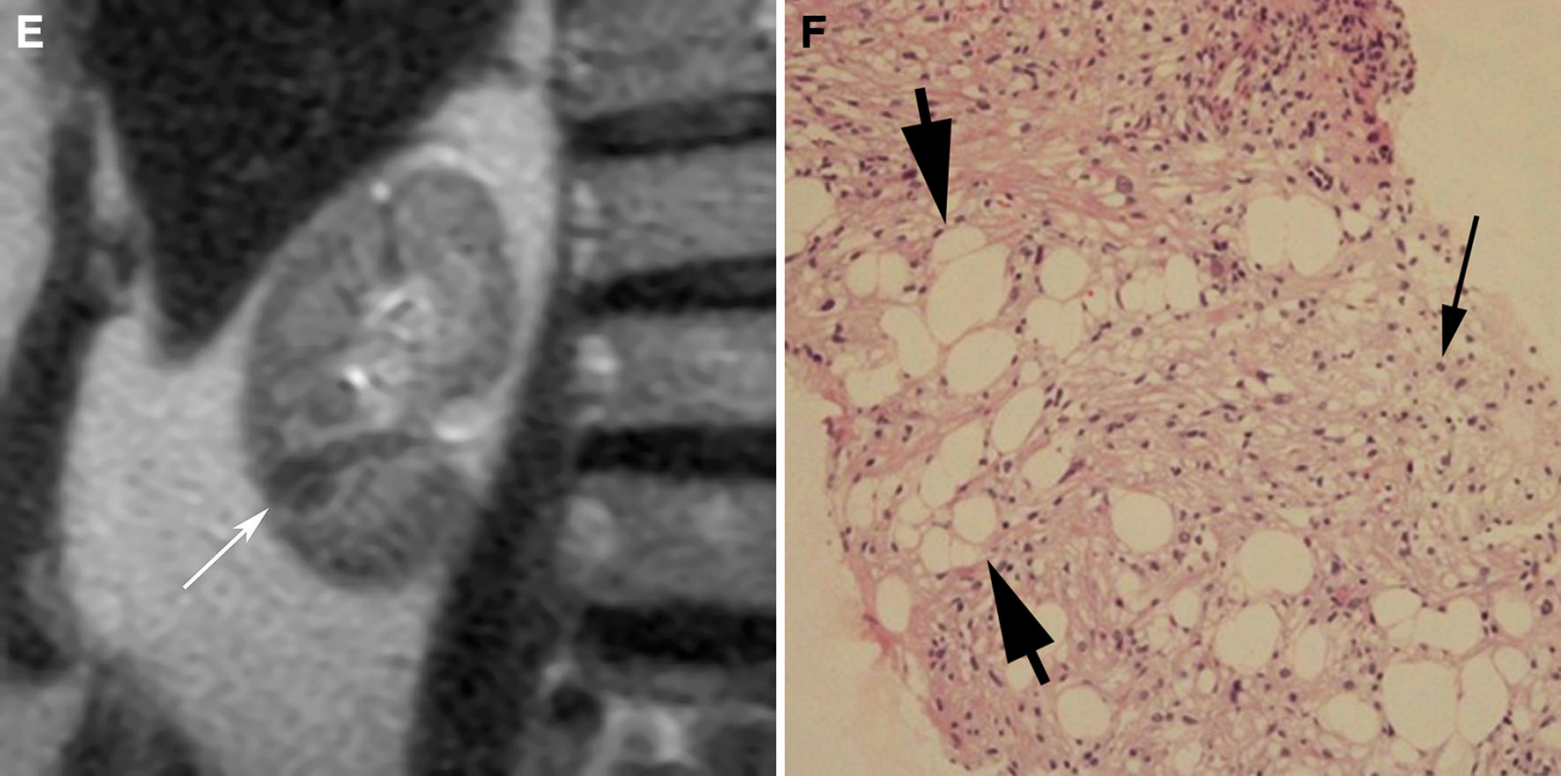
Angiomyolipoma, fat poor, isoattenuating subtype in a 71-year-old woman. Transverse, unenhanced CT (5 mm sections) demonstrates a 1.8-cm isoattenuating right renal mass (*arrows*) with an attenuation of 33HU; no regions measured less than -6 HU (**A**). The mass enhanced to an attenuation of 78HU (**B**). MRI signal intensity of the tumor was 70 on in-phase image (fast spoiled gradient echo, TR:125, TE:4.2) (**C**:* arrows*), and 39 on opposed phase image (fast spoiled gradient echo, TR:160, TE:2.0) (**D**:* arrows*). The mass was hypointense on T2-weighted imaging (single shot fast spin echo, TR:26000, TE:189) (**E**). Because this enhancing renal mass was isoattenuating, T2-hypointense, and suppressed on chemical shift MR imaging, isoattenuating angiomyolipoma was suspected and proven by percutaneous biopsy. Hemotoxlyin-eosin stain specimen (**F**) showed smooth muscle component (*arrow*) and scattered fat cells (*bold arrows*).

Isoattenuating angiomyolipomas by definition contain CT attenuations that are close to renal parenchyma on unenhanced CT (Fig. 4). In our experience, measured attenuations are typically between approximately −10 and 45 HU. These masses by definition contain no regions of fat attenuation at unenhanced CT. This type of angiomyolipoma appears this way because it contains diffuse, scattered fat cells among the smooth-muscle and vessel components, too few in one area to be detected with imaging but sufficient in quantity to lower the overall attenuation relative to hyperattenuating angiomyolipomas [7] (Fig. 4). On MRI, like hyperattenuating angiomyolipomas, they are typically T2-hypointense (due to their smooth muscle component) [55] (Fig. 4). Because isoattenuating angiomyolipomas are so rare, experience regarding their appearance on all MRI pulse sequences is not fully known; they may or may not show signal loss on fat-suppressed pulse sequences depending on the amount and distribution of fat cells in the lesion. However, it should be recognized that unlike hyperattenuating angiomyolipomas, because there are more fat cells, isoattenuating angiomyolipomas typically show chemical shift suppression [55, 62] (Fig. 4). On ultrasound, in our experience, these angiomyolipomas are slightly hyperechoic.

Isoattenuating angiomyolipomas are rare but particularly problematic as there is no specific feature that distinguishes them from RCC and since they are so rare, there is a paucity of literature on how to distinguish the two. Both isoattenuating angiomyolipoma and clear cell RCC can be isoattenuating and enhance at CT [53, 56]. Both can demonstrate suppression at chemical shift MRI [38]. Clear cell RCC may suppress due to the intracytoplasmic lipid in the neoplastic cells; isoattenuating angiomyolipomas may suppress due to the scattered, sparse fat cells. As a result, investigators have attempted to differentiate this type of angiomyolipoma from RCC using different quantitative feature analyses [52–57, 62, 63]. Using CT histograms and chemical shift MRI, isoattenuating angiomyolipoma, relative to RCC, has been shown to exhibit a greater quantitative area of negative CT attenuation and a greater amount of signal loss [52, 62]. However, others have claimed that neither CT histograms nor chemical shift MRI can be used to distinguish them from RCC [53, 56, 64, 65]. Patterns of enhancement have also been analyzed to determine if this type of angiomyolipoma can be differentiated from clear cell RCC. Because most RCC demonstrates early enhancement and rapid washout [66], some have suggested that gradual, or prolonged enhancement on CT is suggestive of angiomyolipoma [52]. However, others maintain that most angiomyolipomas show an arterial-to-delayed enhancement ratio greater than 1.5 [55]. Others have evaluated the usefulness of signal intensity on T2-weighted imaging; fat poor angiomyolipoma, including the isoattenuating type, is virtually always T2-hypointense, and clear cell RCC is typically T2-hyperintense [55, 63]. However, papillary RCC is typically T2-hypointense also [67, 68]. Sasiwimonphan, et al suggested recently that fat poor angiomyolipoma (including the isoattenuating type) could be reliably differentiated from RCC if they were T2-hypointense and demonstrated markedly early enhancement and subsequent washout [55].

Another problem with understanding how to diagnose isoattenuating angiomyolipoma is that previous studies included all fat poor angiomyolipomas and hence included both hyperattenuating and isoattenuating angiomyolipomas [52–54, 56, 65]. Also, since the diagnosis of an isoattenuating angiomyolipoma depends on finding no regions of fat using 1.5–3-mm thin section CT technique, some of the reported angiomyolipomas may not have been fat poor as 5-mm sections were used in some patients [52–54, 56, 65]. If thin, 1.5–3-mm sections had been used, small amounts of fat may have been detected in some lesions. As a result, the frequency of fat poor angiomyolipomas (especially the isoattenuating subtype) would be smaller than what has been reported [mean 6.8 cases/year (20–58 cases/4–7.5 years) in these studies] [52–54, 56, 65]. Although there is no single feature that is diagnostic, an enhancing renal mass that is both T2-hypointense and suppresses on chemical shift imaging (Fig. 4) should prompt consideration of an isoattenuating angiomyolipoma. However, the only nonsurgical way to confirm this diagnosis with confidence is a percutaneous biopsy. Further study will be needed to determine if this combination of features can be used to diagnose an isoattenuating angiomyolipoma with sufficient confidence to avoid a biopsy.

### Angiomyolipoma with epithelial cysts

Angiomyolipoma with epithelial cysts is another extremely rare variant of angiomyolipoma that contains epithelial-lined cysts. They are a type of fat poor angiomyolipoma because they almost always contain few, if any, fat cells [69]. Like classic angiomyolipoma, these lesions are benign and more common in women [7, 8, 69–71]. Although entrapped dilated renal tubules on pathology have been described in classic angiomyolipoma, it is extremely uncommon for classic angiomyolipoma to contain cystic features by imaging; such features were found in only 18 patients among five series [7, 8, 69–71]. In angiomyolipoma with epithelial cysts, the smooth muscle component predominates, however, unlike other types, these lesions contain epithelial cysts and a compact subepithelial stroma. Immunohistochemically, the subepithelial compact stroma and muscle components stain positive for HMB-45, estrogen and progesterone receptors, actin and desmin [7, 8].

**Fig. 5:**
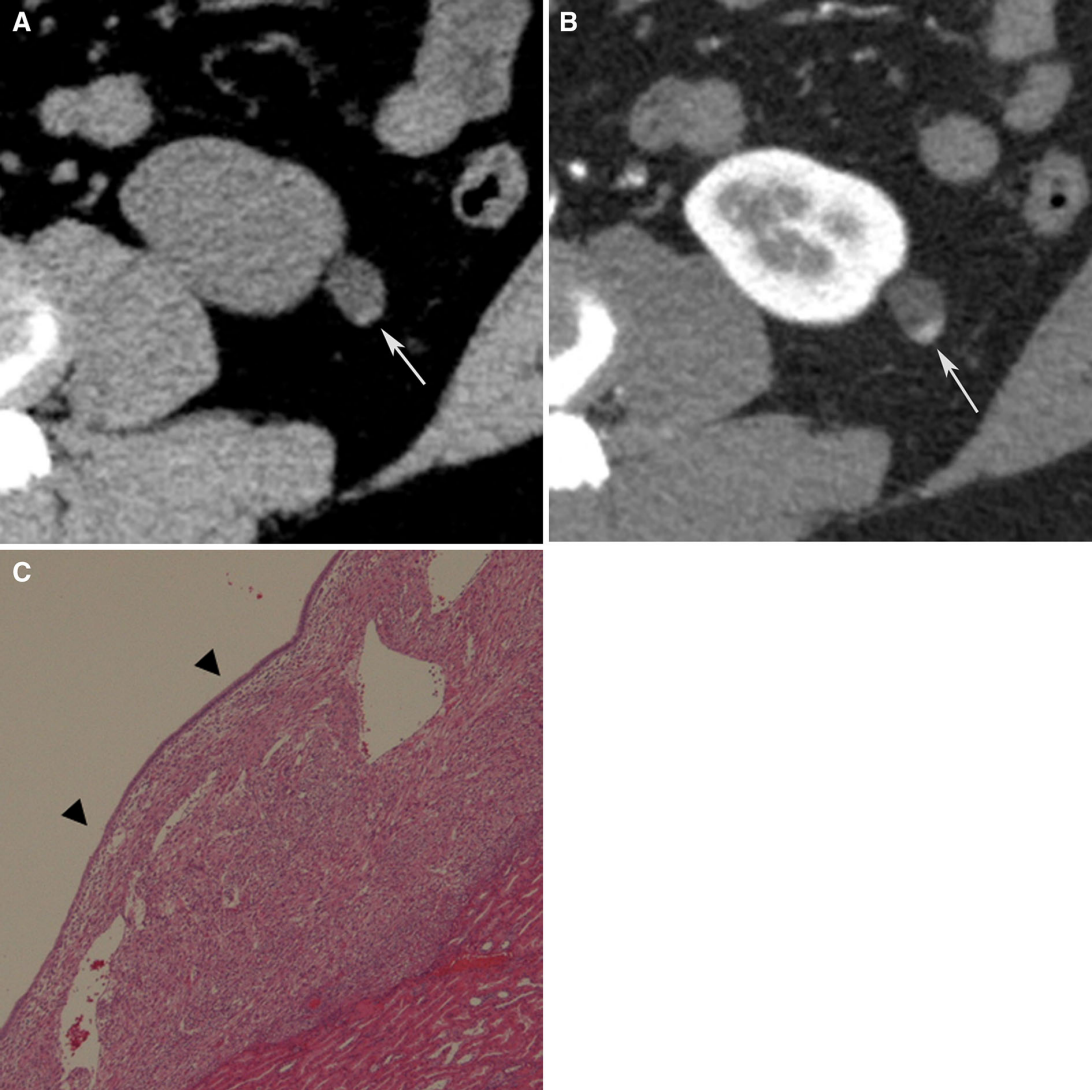
Angiomyolipoma with epithelial cysts in a 60-year-old man. Transverse, unenhanced CT image (1.5 mm sections) shows a 2.0-cm left renal mass with central, hypoaattenuating (5 HU) and a peripheral hyperattenuating (50 HU) components (**A**:* arrow*). The peripheral component enhanced (**B**:* arrow*). This mass was resected during nephrectomy for an ipsilateral renal cell carcinoma. Pathology revealed angiomyolipoma with an epithelial-lined cyst (**C**:* arrowheads*). The cystic area on pathologic specimencorrespond to the low attenuation area seen on CT.

**Fig. 6:**
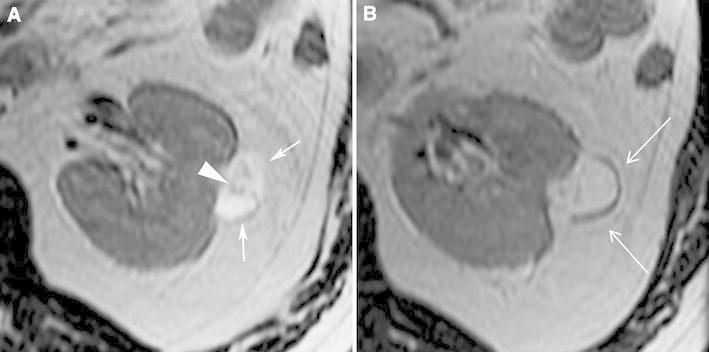
Angiomyolipoma with epithelial cysts in a 46-year-old man. Transverse, T2-weighted MR image (fast spin-echo, TR:4000, TE:92) shows a 2.5-cm multilocular cystic mass with a hyperintense central component, and hypointense wall (**A**, **B**:* arrows*) and septa (**B**:* arrowhead*). Because of suspicion for renal cell carcinoma, this mass was resected. The specimen showed angiomyolipoma with epithelial cysts; the wall contained smooth muscle component.

Imaging findings of angiomyolipoma with epithelial cysts are not fully known. One report described one that was hyperattenuating on unenhanced CT (Fig. 5), T2-hypointense (both due to its smooth muscle component) (Fig. 6), and contained a small cyst; the non-cystic portion of the tumor-enhanced homogeneously [69]. Another report described a multilocular cystic mass that contained cystic components separate from the smooth muscle component [70] (Fig. 6). Since cystic components are a rare manifestation of angiomyolipoma, RCC should be considered when a solid mass contains cystic components. However, if the rest of the mass is hyperattenuating on unenhanced CT (Fig. 5), T2-hypointense (Fig. 6), and homogeneously enhancing, an angiomyolipoma with epithelial cysts should be considered also. Indeed, this entity should be added to the differential diagnosis of a multilocular cystic mass in an adult along with a multilocular cystic RCC, multilocular cyst, cystic nephroma, and a mixed epithelial and stromal tumor (MEST) [70]. Biopsy may not be diagnostic of a multilocular cyst or a cystic nephroma. However, because both angiomyolipoma with epithelial cysts and MEST may stain positive for desmin or actin, and RCC does not, percutaneous biopsy may be helpful in diagnosing a benign neoplasm [70, 72].

## Epithelioid angiomyolipoma

Epithelioid angiomyolipoma is an extremely rare type, first described by Eble et al. [9] in 1997, that is composed of numerous atypical epithelioid muscle cells. Most of these tumors contain few or no fat cells [9, 73, 74]. Both genders are equally affected, and the mean age of diagnosis is 38 years [1]. Unlike all other angiomyolipomas, the epithelioid type is potentially malignant and can be locally aggressive and metastasize [1]. Approximately one-third demonstrate local extension or distant metastases at presentation; the larger the tumor, the more likely it will spread [75]. Histologically, these tumors can resemble and be misdiagnosed as sarcomatoid or high grade RCC [1]. However, an epithelioid angiomyolipoma can be differentiated from RCC by the presence of immunohistochemistry markers such as melanosome-associated proteins (HMB-45 antigen, melan-A) and smooth muscle markers (HHF-35, SMA, and caldesmon) [9, 73]. In addition, epithelial tumor markers that are typically positive in RCC such as epithelial membrane antigen (EMA) and cytokeratins are negative in epithelioid angiomyolipoma [1].

**Fig. 7:**
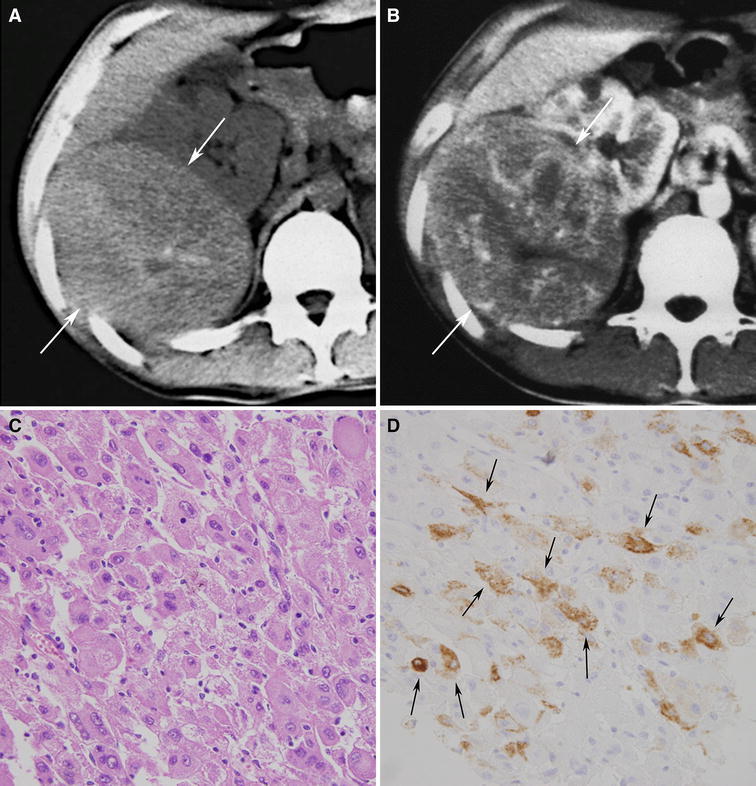
Epithelioid angiomyolipoma in a 21-year-old man. Transverse, unenhanced CT (5 mm sections) shows a 12.0 cm hyperattenuating right renal mass (**A**:* arrows*) that enhanced heterogeneously (**B**:* arrows*). No regions of fat attenuation could be identified. Hematoxylin–eosin staining specimens show pleomorphic tumor cells with large hyperchromatic nuclei and abundant eosinophilic cytoplasm (**C**). The tumor cells were was positive for HMB-45 (**D** *arrows*).

**Fig. 8:**
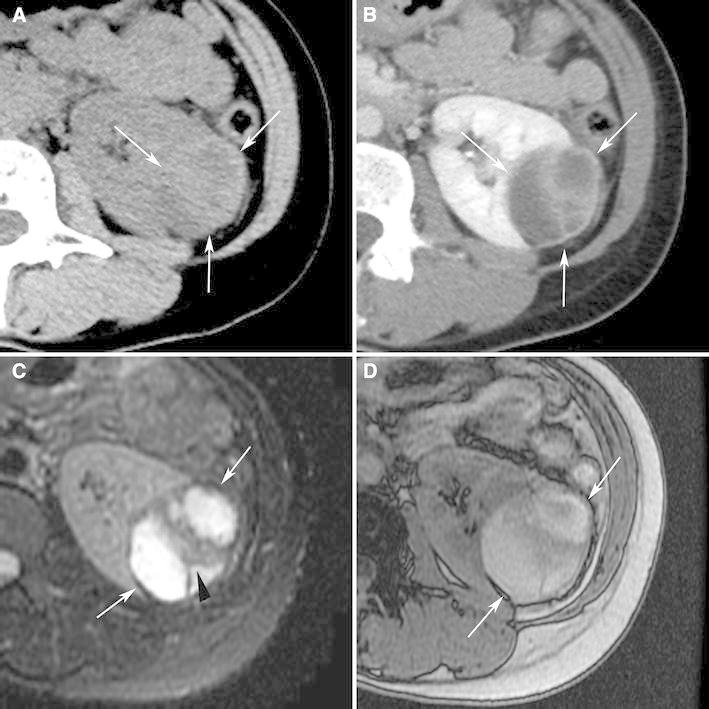
Epithelioid angiomyolipomae in a 40-year-old woman. Transverse, unenhanced CT (5-mm sections) (**A**) and enhanced CT (**B**) demonstrates a 5.0-cm multilocular cystic mass in the left kidney. Both the wall and septa (*arrows*) were hyperattenuating (48 HU) and enhanced. On transverse, T2-weighted MR image (fat-saturated fast spin-echo, TR:3800, TE: 89) (**C**), the wall (*arrows*) and septum (*arrowhead*) of the multilocular cystic mass appeared hypointense. On a transverse, T1-weighted MR image (fast spoiled gradient echo, TR: 150, TE:2.3) (**D**), the cystic component was hyperintense (*arrows*). The mass was considered a Bosniak Category 3 lesion and resected due to its appearance. The hyperintense cystic component corresponded to hemorrhage at pathology.

Radiologically, epithelioid angiomyolipomas typically present as large masses with intratumoral hemorrhage and necrosis [76–81] (Fig. 7). They average 7-cm in size and are typically much larger than fat poor angiomyolipomas [80]. They, along with triphasic angiomyolipomas, may be accompanied also by a spontaneous perirenal hematoma [80, 81]. Small foci of fat can be detected with CT or MRI in some patients [80]. Most epithelioid angiomyolipomas are hyperattenuating on unenhanced CT (typically more than 45 HU) and T2-hypointense due to their epithelioid muscle component [81]. A recent study reported they can present as heterogeneously or homogeneously enhancing solid masses or as multilocular cystic masses [81] (Figs. 7, 8). The amount of described enhancement was variable. Multilocular cystic epithelioid angiomyolipoma tends to exhibit more hemorrhage than is typically seen with cystic RCC; sometimes it is massive and can be a clue to the diagnosis [81].

The preoperative distinction between epithelioid angiomyolipoma and RCC may not be critical as both lesions are treated with surgical resection. However, the mTOR pathway was recently found to be activated in epithelioid angiomyolipoma [82], and some studies have reported that mTOR inhibitors, such as sirolimus or temsirolimus, may represent a better treatment option for patients with epithelioid angiomyolipoma [83, 84]. Hence the image-based pre-operative diagnosis of this type of angiomyolipoma may become important in the future.

## Angiomyolipoma in tuberous sclerosis complex

**Fig. 9:**
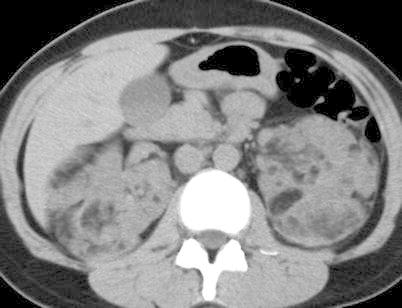
Angiomyolipoma in a 32-year-old woman with tuberous sclerosis complex. Transverse, unenhanced CT (5-mm sections) shows multiple bilateral renal masses each containing fat attenuation (less than −10 HU) diagnostic of angiomyolipomas.

Angiomyolipomas are observed in 55%–75% of patients with TSC; most form by the third decade [85]. Relative to sporadic angiomyolipoma, both genders are affected equally. Angiomyolipomas in TSC typically present at a younger age, are more often multiple, larger, and almost always bilateral (Fig. 9).

Most angiomyolipomas in TSC are histologically identical to the classic type, however, like other sporadic forms, they may contain few to no fat cells. Fat poor angiomyolipomas have been reported to occur in over one-third of patients with TSC. Fat poor angiomyolipomas in TSC appear the same as those presenting sporadically except they tend to be larger [35]. Since RCC can occur in patients with TSC, masses that do not contain visible fat may require a percutaneous biopsy or close follow-up [40]. Epithelioid angiomyolipoma and angiomyolipoma with epithelial cysts are also both seen in patients with TSC [9]. Angiomyolipomas in patients with TSC are more likely to have an epithelioid component or contain epithelial cysts compared to angiomyolipomas found sporadically [86].

Relative to the general population, angiomyolipomas in patients with TSC are more likely to need some form of treatment. Angiomyolipomas in TSC tend to grow and be more symptomatic [87]. One study reported TSC-associated angiomyolipomas grew an average of 1.25 cm/year compared to an average growth rate of sporadic ones of only 0.19 cm/year [40]. Recurrent angiomyolipoma bleeding may occur in as many as 43% of patients with TSC where as sporadic angiomyolipomas typically don’t rebleed [88, 89]. The presence of multiple angiomyolipomas often leads to multiple bleeds, and the need for repeated treatments. To avoid the need for repeated surgery, transcatheter embolization (TCE) is the preferred treatment in patients with TSC with angiomyolipomas that have bled. Although TCE is effective in controlling hemorrhage in the acute setting, it may not prevent rebleeding and appears to be of limited value in the long-term [47]. The mTOR inhibitor sirolimus, by inhibiting the activation of the mTOR pathway, has been found to be effective in preventing tumor growth and re-bleeding in patients with TSC [90].

## Angiomyolipoma in lymphangioleiomyomatosis

Renal angiomyolipomas may also occur in patients with lymphangioleiomyomatosis (LAM), a rare disease characterized by proliferation of atypical smooth muscle-like cells with associated cystic changes. LAM typically presents with symptoms related to the destructive cystic changes in the lungs. The pulmonary disease is progressive and may result in pneumothoraces, chylous pleural effusions, and respiratory failure. LAM occurs sporadically or in association with TSC [11]. Sporadic LAM affects 1 in 400,000 adult females; in TSC, LAM occurs in 30%–40% of adult females [11] and rarely in males and children. Although the principal clinical manifestations are derived from pathology in the lungs, LAM is a multisystem disorder that in addition to renal angiomyolipomas, causes abdominal lymphadenopathy, chylous ascites, and large cystic lymphatic masses called lymphangioleiomyomas [11, 12, 91, 92]. LAM is also associated with an increased frequency of meningioma. While inherited mutations of the TSC-1 or TSC-2 genes cause TSC, acquired (somatic) mutations of either gene are associated with sporadic LAM [11].

The prevalence of renal angiomyolipomas in sporadic LAM is 40%–54% when patients with the disease are evaluated with abdominal CT [11, 12]. According to a study that evaluated abdominal findings in 80 patients with LAM not associated with TSC, renal angiomyolipomas were observed in 43 (54%) patients, enlarged lymph nodes in 31 (39%), and lymphangioleiomyomas in 13 (16%) [12]. In the same study, CT depicted 76 renal angiomyolipomas in 40 patients; masses were single in 23 (57%) patients and multiple in 17 (43%) patients. Angiomyolipomas were 0.2–9.0 cm in diameter (mean, 1.3 cm); 55 (72%) measured less than 1.5 cm in diameter, while 44 (58%) measured less than 1.0 cm in diameter. This study also reported a patient with a fat poor angiomyolipoma diagnosed by biopsy. Although there are few reports of angiomyolipomas in patients with LAM not associated with TSC, they are typically smaller, less frequently bilateral, and less prone to bleeding than those found in patients with TSC [11]. While one study reported an epithelioid angiomyolipoma in LAM [93], to our knowledge, an angiomyolipoma with epithelial cysts has not been reported in a patient with LAM not associated with TSC. The European Respiratory Society guidelines recommend that patients with unilateral angiomyolipomas, no clinical features of TSC, and no pulmonary symptoms, should be evaluated for LAM by undergoing HRCT of the chest [18]. In patients with bilateral angiomyolipomas, evaluation for TSC is recommended via a thorough personal and family history for manifestations of TSC. If TSC is found, evaluation for LAM with HRCT of the chest is recommended [11].

As with sporadic angiomyolipoma, the guidelines for patients with LAM also recommend that asymptomatic small (<4 cm) renal angiomyolipomas be followed yearly with ultrasound [11]. Larger angiomyolipomas or small ones with aneurysms 5 mm or larger in diameter are thought to be at an increased risk for bleeding, and hence followed with ultrasound twice yearly to evaluate for growth. Treatment by renal arterial embolization or partial nephrectomy can be considered when an angiomyolipoma bleeds. Recently, two prospective open-label clinical trials in patients with LAM and angiomyolipoma found that the mTOR inhibitor sirolimus reduced angiomyolipoma volume [90, 94]. However, mTOR inhibitors are not recommended as first-line therapy because their ability to prevent bleeding has not been evaluated and side-effects are common [90, 94]. Furthermore, the relative risks and benefits of treatment with sirolimus have not been compared to renal arterial embolization or partial nephrectomy. Embolization is generally recommended, with partial nephrectomy reserved for masses in which a malignancy is considered. Sirolimus may be considered in patients with symptomatic angiomyolipoma not amenable to embolization or surgery.

## Conclusion

Our understanding of the pathology, radiology, and clinical behavior of angiomyolipoma has matured well beyond the original “classic” descriptions. The variable and heterogeneous nature of this neoplasm now considered among the family of PEComa, is indeed important to understand in clinical practice. As radiologists, knowledge of the various types, their imaging appearance, and how they are classified will help in both diagnosis and treatment. Although the detection of fat is a well-established diagnostic imaging feature of classic angiomyolipoma, a hyperattenuating appearance on unenhanced CT and a T2-hypointense appearance at MRI both correspond to the smooth muscle component, are important diagnostic clues to the types of angiomyolipomas that contain few or no fat cells. Because malignancies can also demonstrate these imaging characteristics, percutaneous biopsy is recommended for small (less than or equal to 3 cm) hyperattenuating, T2-hypointense, enhancing renal masses so that unnecessary surgery can be avoided. When hyperattenuating, T2-hypointense, enhancing renal masses are larger, or there is evidence of massive hemorrhage, proceeding directly to surgery may be appropriate, both to prevent further bleeding and because both renal cancer and epithelioid AML are more likely.
